# Multiple entries and exits and other complex human patterns of insecticide-treated net use: a possible contributor to residual malaria transmission?

**DOI:** 10.1186/s12936-017-1918-5

**Published:** 2017-07-03

**Authors:** Steven A. Harvey, Yukyan Lam, Nina A. Martin, Maribel Paredes Olórtegui

**Affiliations:** 1 Johns Hopkins Bloomberg School of Public Health, Baltimore, MD USA; 2 Asociación Benéfica PRISMA, Iquitos, Peru

## Abstract

**Background:**

Increased insecticide-treated net (ITN) use over the last decade has contributed to dramatic declines in malaria transmission and mortality, yet residual transmission persists even where ITN coverage exceeds 80%. This article presents observational data suggesting that complex human net use patterns, including multiple entries to and exits from ITNs by multiple occupants throughout the night, might be a contributing factor.

**Methods:**

The study included dusk-to-dawn observations of bed net use in 60 households in the Peruvian Amazon. Observers recorded number of net occupants and the time and number of times each occupant entered and exited each net. The study team then tabulated time of first entry, total times each net was lifted, and, where possible, minutes spent outside by each occupant.

**Results:**

The sample included 446 individuals and 171 observed sleeping spaces with nets. Household size ranged from 2 to 24 occupants; occupants per net ranged from 1 to 5. Nets were lifted a mean 6.1 times per night (SD 4.35, range 1–22). Observers captured substantial detail about time of and reasons for net entry and exit as well as length of time and activities undertaken outside.

**Conclusions:**

These findings suggest that the ITN use patterns observed in this study may contribute to residual transmission. As a result, respondents to net use surveys may truthfully report that they slept under a net the previous night but may not have received the anticipated protection. More research is warranted to explore the impact of this phenomenon. Concurrent entomological data would help assess the magnitude of the effect.

## Background

**Fig. 1:**
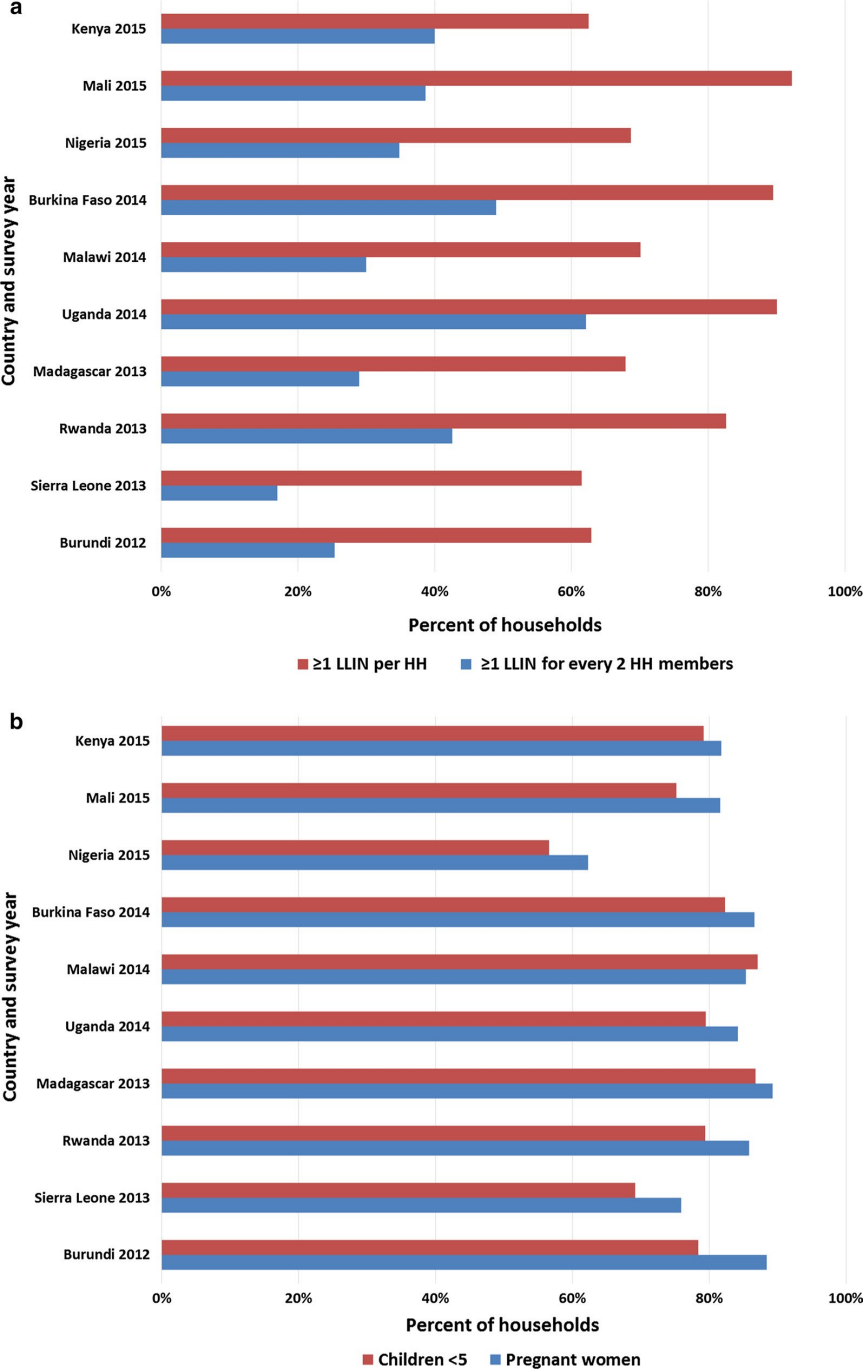
**a** Percent of households with ≥1 LLIN per household and with ≥1 LLIN per two household members; **b** Percentage of children <5 years and pregnant women who slept under a net the night prior to the survey in households with at least one LLIN

Insecticide-treated nets (ITNs) are a critical tool in malaria control [1]. With international donor support, malaria-endemic countries have distributed nearly 1.3 billion ITNs worldwide since 2004, 1.1 billion in sub-Saharan Africa [2]. The World Health Organization (WHO) estimates that about 67% of the world’s at-risk population now has access to an ITN, a dramatic increase from under 2% in 2000. WHO further estimates that 82% of those with access sleep under a net, although some studies suggest that actual use may be much lower [3–6]. Figure 1a, b illustrate reported net access and use by children under 5 years old and pregnant women from ten African countries [7–16].

Increased access and use have significantly reduced malaria transmission: WHO’s Global Malaria Programme estimates that between 2000 and 2015, annual incidence fell by 37% and deaths by 60% [3]. Residual transmission persists, however, even where long-lasting insecticidal net (LLIN) coverage exceeds 80%. Contributors include insecticide resistance, changes in vector biting patterns, a shift to more exophagic vector species, and human social activities during peak biting periods [17–20]. This paper suggests another possible contributor: complex human net use patterns not captured by existing measurement methods.

Data like those in Fig. 1 come from malaria indicator surveys (MIS) or similar health surveys. They are typically based on a two-question sequence for each net: (1) “Did anyone sleep under this mosquito net last night?”; and, (2) “Who slept under this mosquito net last night?” [7–16]. An affirmative answer to question 1 however, does not necessarily mean that the individual or individuals identified in question 2 slept under the net *throughout* the night.

This paper presents data showing that users of a single net may go to bed at different times, with those retiring later remaining exposed to infective bites. Further, different users may enter and exit multiple times during the night, exposing themselves to infective bites outside while simultaneously allowing mosquitoes to enter and bite others inside. These multiple entries and exits, particularly late at night when the person entering or exiting is not fully awake, could leave the net inadequately secured, thus providing an additional route for mosquitoes to enter. Finally, the data show that some individuals spend only part of the night under the net or sleep partially inside and partially outside.

The data are based on nighttime observations of net-using households in a malaria-endemic region of the Peruvian Amazon. While collected before the era of LLINs and mass distribution, they remain relevant to the issue addressed in this paper: they demonstrate that self-reported net use is not necessarily equivalent to spending the entire night protected, and that multiple entries and exits may compromise a net’s protective efficacy. To the authors’ knowledge, the type of data described here—dusk-to-dawn observations of individual net use in households—is unique and reveals details not measurable by other methods.

## Methods

### Data collection

Between 4 February and 21 June, 2000, four observers, including authors SH and MPO, conducted 60 direct night-time observations of households in four semi-rural villages: El Manantial, San Anselmo, Villa Buendía, and Santa Catalina (all pseudonyms). All are located within 30 km of Iquitos, the Peruvian Amazon’s largest city. Principal economic activities are subsistence and small-market agriculture supplemented by fishing, hunting, artisanal charcoal production, and similar activities. Three of the villages had no electricity; in the fourth, 25–30% of households had at least provisional access.

Each observation occurred over a 12- to 14-h period between approximately 17:30 and 07:30. Observers collected both structured and unstructured data. Upon arrival, the observer asked a family member, usually the female head of household, for a tour of the house. Using a ten-item structured form, the observer recorded age, gender and relationship of all household members, number and type of sleeping spaces and nets, which household members slept in which sleeping spaces, and other pertinent information. For the rest of his or her stay, the observer took handwritten, unstructured notes on all household activities at 5-min intervals, including the whereabouts of each household member. Since most households consisted of raised wooden platforms with thatched roofs and few rooms or interior dividers, observers could usually see most household activities from a single vantage point.

Community health workers in each village helped recruit households for observation. Selection was purposive and attempted to maximize diversity of socio-economic status, household size, proximity to the village centre, and proximity to rivers.

At the time observations were conducted, their main objective was not to record net entry and exit per se, but to document the extent to which area residents used nets, types of beds and nets used, manner of use, times at which people went to bed and woke up, and other common social, religious and economic activities that might influence protective effect [21, 22]. However, as net entry and exit were recorded fairly systematically, it is useful to highlight the unexpected amount of entry and exit activity observed.

Reactivity, defined as a situation in which someone being observed changes his or her behaviour due to an observer’s presence, is a frequently expressed concern in direct observation [23]. To minimize reactivity, observers were instructed not to mention bed nets specifically when introducing the study but to stress the aim of better understanding living conditions that might affect malaria risk and prevention. Observers were also instructed to station themselves as inconspicuously as possible within the household and to limit contact with household members. To measure potential reactivity, observers recorded all instances of interaction between themselves and household members. Reactivity results are reported elsewhere [24].

### Data analysis

The unit of analysis for this paper is the sleeping space. The term ‘sleeping space’ rather than ‘bed’ is used here because only a minority of people observed slept on frame-and-mattress beds. Most slept on a wooden platform, directly on a split bamboo or wooden plank floor or, occasionally, in a hammock or directly on the ground.

Handwritten observation notes were transcribed into MS Word, then imported into the qualitative data analysis program ATLAS.ti [25]. Codebook development began using open coding as defined in Grounded Theory [26]. With the reading of each transcript, codes were added to the codebook as new themes emerged. By the 15th transcript, the codebook contained 73 thematic codes and no new themes were emerging. At that point, the initial 15 transcripts were re-coded using the complete 73-code scheme. The same complete scheme was then applied to the remaining 45 transcripts. To track the time at which each activity took place, 20 time-based codes were then overlaid, one for each half-hour from 17:00 to 22:00 and one for every hour from 22:00 to 07:59, a less active period.

Data presented here draw primarily on two codes: ‘enter net’ and ‘exit net’. Using a standardized 14-h observation interval from 17:00 to 06:59, these codes were applied to identify the number of occupants, the number of entries and exits, and the time at which each entry or exit occurred for each net. The coders iteratively coded and reviewed, checking one another’s work to ensure comprehensiveness and accuracy. The code ‘sleep without net’ was used to track participants who used a net for only part of the night or were only partially covered (e.g., with arm or leg protruding). ‘Get up at night’ was used to examine reasons for exiting the net and the amount of time spent outside if these were possible to ascertain from the observation notes.

An Excel spreadsheet was used to calculate total entries and exits for each net. Stata 14 was used to calculate sample means and standard deviations for entries and exits overall and stratified by number of occupants and number of different age groups per net [27]. For the latter, the sample was divided into five standardized age groups: <1, 1–5, 6–14, 15–49, and 50+ years. The Shapiro–Wilk test revealed that these variables were not normally distributed, so Spearman’s correlation coefficient was calculated to assess correlation of entries and exits with both number of people and number of age groups sharing a net [28].

### Entomological context of exposure risk

Basic entomological data about *Anopheles darlingi*, the Amazon’s principal malaria vector, served as a parameter for analysis. A highly efficient vector, *An. darlingi* adapts feeding behaviour to changing human ecology. Studies report different peaks, but concur that biting begins around dusk [29]. Since sunset occurs no later than 18:15 in the study area, exposure was assumed to begin at 18:30 local time [30].

### Ethical review

The study was approved by the Johns Hopkins Bloomberg School of Public Health Institutional Review Board in Baltimore, Maryland, USA and the Ethics Committee of the *Asociación Benéfica PRISMA* in Lima, Peru. Written informed consent was obtained from all participants in accordance with the requirements of both committees.

## Results

The 60 households observed ranged from 2 to 24 occupants, including nuclear and extended family and occasionally, hired labourers. Ten households (16.7%) had at least ten occupants; 26 (43.3%) had between six and nine. Overall, the sample included 446 individuals and 201 sleeping spaces. Of the spaces observed, only five (2.5%) lacked any type of net. Use could not be confirmed for ten spaces (5%), usually because the occupant was travelling with their net.

From the 201 sleeping spaces in the overall sample, 30 (14.9%) were excluded from analysis for reasons described in Table 1. Table 2 provides descriptive statistics for the sample before and after exclusion. Of the 171 post-exclusion sleeping spaces, 102 (60%) had one or two occupants, while 63 (37.1%) had three or four. Five spaces (2.9%) had five occupants each.

**Table 1:** Reasons for exclusion and sleeping spaces affected

Reason for exclusion	No. (%) sleeping spaces excluded
Sleeping space not used on night of observation	12 (5.9)
Observer only able to track 4 out of 10 sleeping spaces in one household due to large number of occupants (24) and sleeping spaces (10)	6 (3.0)
Sleeping space had no bed net	5 (2.5)
Observer ended observation at 21.50 (1 household)	4 (2.0)
No adults present (1 household)	2 (1.0)
Sleeping space located in chicken coop; observer had no access	1 (0.5)
Total	30 (14.9)

**Table 2:** Sample characteristics and age group in years

Sample characteristics				Mean	SD	Min, max
Occupants per household (n = 60 HH)				7.43	3.67	2, 24
Occupied sleeping spaces per household overall (n = 189*)				4.22	2.10	1, 10
Occupied sleeping spaces per household after exclusions (n = 171*)				3.99	1.84	1, 10
Occupants per sleeping space with a net (n = 171 nets*)				2.32	1.09	1, 5
Age (years) before exclusion (n = 446 individuals)				21.5	18.1	2 months, 80
Age (years) after exclusion (n = 397 individuals)				21.6	18.5	2 months, 80

Age group (years)	<1	1–4	5–14	15–49	50+	Total
n (overall)	13	56	138	194	45	446
% (overall)	2.9	12.6	30.9	43.5	10.1	100
n (after exclusion)	12	51	126	166	42	397
% (after exclusion)	3.0	12.9	31.7	41.8	10.6	100

* 201 total sleeping spaces minus 12 unoccupied (Table 1) = 189 occupied sleeping spaces observed minus 18 excluded for other reasons (Table 1) = 171 observed sleeping spaces included in final analysis

**Fig. 2:**
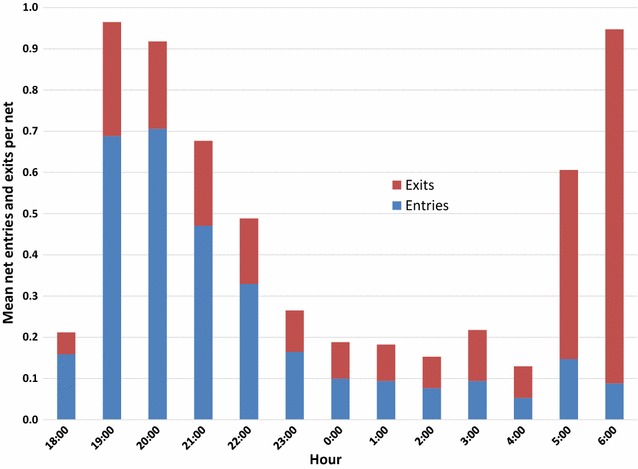
Mean net entries and exits per hour for 171 sleeping spaces

Nets were lifted a mean 6.1 (SD 4.35) times over the 14-h observation interval. The number lifts per net ranged from 1 to 22. Figure 2 presents mean entries and exits per hour for the 171 sleeping spaces. As one might expect, the greatest number of net entries occur between 19:00 and 22:59 and the greatest number of exits between 05:00 and 06:59 with much less activity in between.

**Fig. 3:**
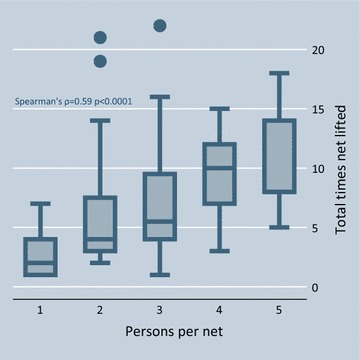
Number of times net lifted per night by number of net occupants

**Fig. 4:**
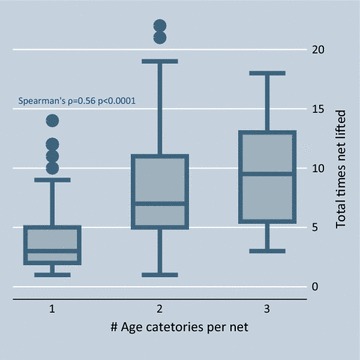
Number of times net lifted by number of age groups sleeping in the net. There were five age groups represented in the sample: (1) less than 1 year, (2) 1–5 years, (3) 6–14 years, (4) 15–49 years, and (5) over 50 years, but a maximum of three different age groups represented in any one net

Fourteen of the 171 nets were lifted only once: in 13 cases the sleeping space was occupied by a single individual who entered the net at some point during the night but had not yet exited by 06:59; in one case the sleeping space was shared by a 49-year old woman, her 10-year old grandson and 12-year old granddaughter. The net lifted 22 times belonged to a sleeping space shared by three individuals: a husband and wife, ages 22 and 24, respectively, and their 2-year old daughter. Apart from this extreme case, observers documented 36 nets (21%) lifted ≥10 times and seven (4%) lifted ≥15 times. Number of lifts was positively correlated with both number of occupants (Spearman’s ρ = 0.59 p < 0.0001) and number of age groups (ρ = 0.56 p < 0.0001) sharing the net. Figures 3 and 4 illustrate this relationship.

**Fig. 5:**
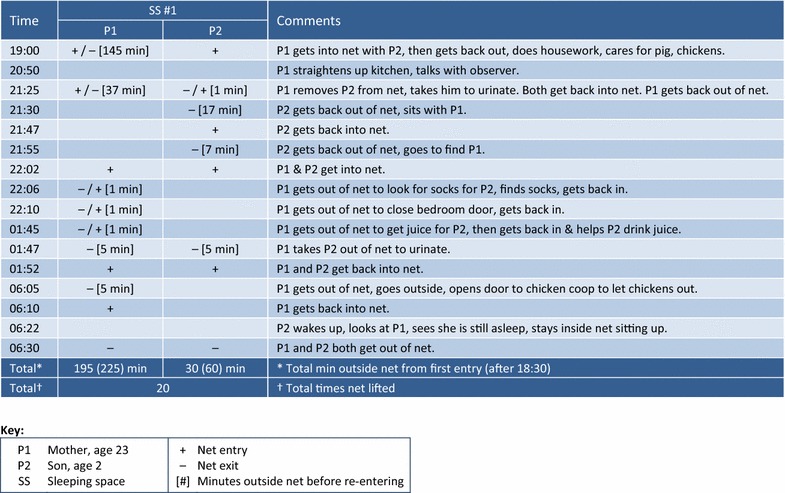
Villa Buendía 13 March 2000, household 02: entries, exits, time outside net (1 sleeping space, 2 people)

**Fig. 6:**
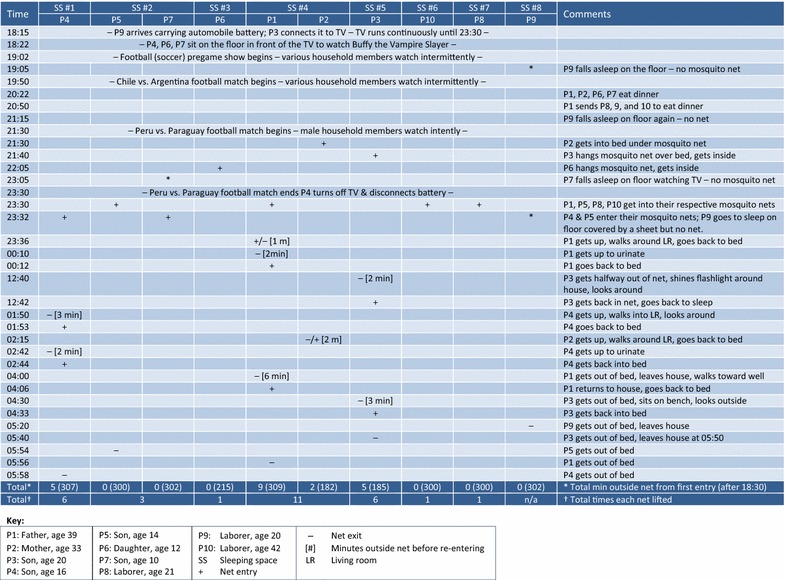
Manantial 29–30 March 2000, household 20: entries, exits, time outside net (8 sleeping spaces, 9 people)

**Fig. 7:**
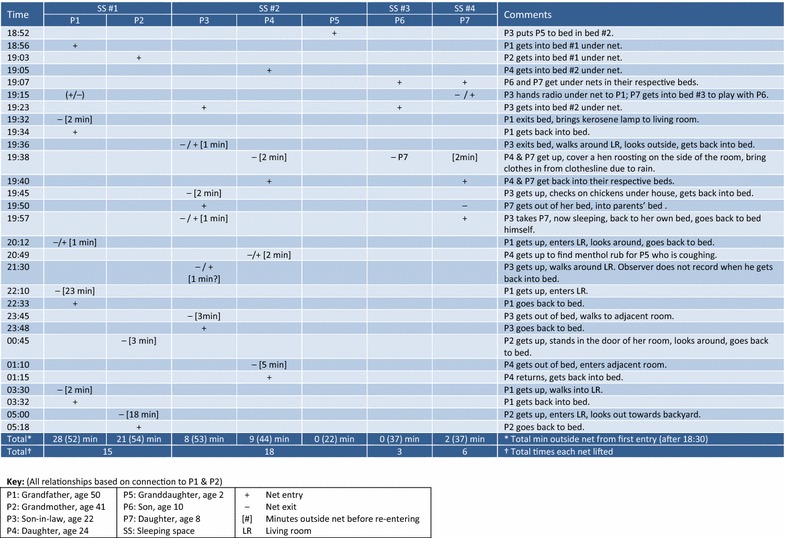
Villa Buendía 13 Mar 2000, household 14: entries, exits, time outside net (4 sleeping spaces, 7 people)

Observers captured substantial detail about reasons for entering and exiting nets, times at which entries and exits occurred, and sometimes time spent and activities undertaken outside. Figures 5, 6, and 7 illustrate scenarios from three different households, representing different types of activities that can delay or interrupt net use. The key below each figure includes occupant relationship, age, and gender, plus symbols for entry (+), exit (−), minutes spent outside the net [## min] and other abbreviations. Sleeping spaces are indicated by number (SS #1, SS #2, etc.).

Figure 5 tracks activity in one net occupied by a single mother and her 2-year old son. The two slept directly on a split bamboo (*pona*) floor with the net hanging over it. The house was built on stilts about 1 m off the ground to protect against flooding in rainy season. It was located in Villa Buendía, a rural community about 1 h by bus from Iquitos followed by a half-hour walk from the road. After entering the net for the first time at 19:00, the mother (P1) spent 195 min outside it during the night, caring for farm animals and doing housework (19:00–21:25; 145 min), relaxing and taking care of her son (21:25–22:02; 37 min), then getting in and out several times for short intervals between 22.06 and 06.30. Adding the 30 min between 18:30 and the mother’s first entry at 19:00, her total theoretical exposure time was 225 min. The son’s theoretical exposure time was 60 min: 30 prior to entering at 19:00, followed by one 17-min interval (21:30–21:47) and three shorter intervals totalling 13 min. Between mother and son, the net was lifted 20 times from 19:00 to 06:30 (instances where both get in or out at the same time are counted only once).

Figure 6 shows a household located in Manantial, a neighbouring village about 40 min by road plus a 15-min walk from Iquitos. This ten-member household had eight sleeping spaces: one wooden platform bed (SS #1), one on a wood plank floor (SS #6), and the rest on the *pona* floor. Sleeping spaces 1–6 had nets; 7 and 8 did not. The main theoretical source of exposure in this household is not multiple net entries and exits, but extended unprotected intervals before entering a net, mostly due to television watching from 18:20 to 23:30. Napping or resting outside a net by individuals who sleep under a net at other times of night might also contribute.

Figure 7 shows a seven-member household located in Villa Buendía and built on stilts. There is minimal exposure prior to net entry: all occupants were inside their nets by 19.07. The nets on sleeping spaces 3 and 4 were lifted 3 and 6 times, respectively; those on spaces 1 and 2 were lifted 15 and 18 times. Most exits were for short intervals and routine activities: passing a radio to another family member, bringing a kerosene lamp from one room to another. A thunderstorm during this observation also contributed: a mother (P4) and daughter (P7) get up to bring clothes in from the rain; a father (P3) gets up to check on the family’s poultry; the same daughter (P7) crawls into her parents’ bed when the thunder gets loud, her father (P3) takes her back to her own bed once she falls back to sleep. Overall time outside a net is less than 1 h per person during anopheline biting hours.

Observers recorded six instances of people sleeping outside a net for part of the night. In some cases the individual was sleeping on a chair, hammock or bench before entering a net. In others, the individual spent some time asleep in bed before lowering the net or slept with part of his or her body inside and part outside the net. Field notes provide a few illustrative examples:

P1 (29-year old father) comes back carrying P7 (5-month old son) asleep in his arms. P1 puts P7 into bed 4. Net on bed 4 is not lowered until 23:15, so P7 is sleeping outside a net for 3 h. P1 goes to sleep in bed 4, P8 goes to sleep in bed 5, both of them outside their nets (HH44, 04 February 2000, 20:12–20:15).

P15 (6-year old granddaughter) goes to sleep in hammock; P5 (28-year old daughter) puts P14 (1-year old granddaughter) to sleep in bed 1; both lie down inside net; P13 (4-year old granddaughter) who was sleeping in this bed is now awake and sitting on the edge of the bed outside the net (HH27, 16 February 2000, 20:40).

P7 says to P4, who is half-in and half-out of the net, “be careful, you’re going to let mosquitoes get in.” (HH43, 04 April 2000, 19:15).

Of the 171 sleeping spaces with nets included in the analysis, 55.5% were nets observed to be secured by occupants tucking net edges under mattresses or using clothing or rags to keep the edges in contact with the floor in cases where the net was hung directly over the floor. The remaining 45.5% were left unsecured. Again, field notes provide a few illustrative examples:

Mosquito net partially lowered but unsecured at bottom, hanging over bed with one corner raised about 20 cm where mother got out a few minutes ago (HH01, 04 February 2000, 19:29).

Mother, 45, puts son, 2, to sleep in bed 3. 33-year old man leaves house for 2 min, comes back, goes to sleep in bed 2. 25-year old son goes to sleep in bed 1. None of the nets are secured. (HH19, 27 March 2000, 20:03–20:07).

Children have fallen asleep on floor, 50-year old woman has fallen asleep in hammock. Friend, age 30, is sleeping by himself in bed 6, does not put up his net until 21:10 and does not secure edges. Son, age 17, sleeps by himself in bed 3; double-ply cotton net up since 20:08; he secures it by tucking it underneath the mattress (HH24, 19 June 2000, 21:00).

## Discussion

### Limits to the protective efficacy of ITNs

Despite their effectiveness and growing ubiquity, there are several limitations to the protection an ITN can provide. One is access: despite distribution of over a billion ITNs in sub-Saharan Africa, many households and individuals still have no net at their disposal [2, 3]. A second is failure to use an available net. Malaria indicator surveys (MIS) appear to show that most people with net access use a net, but social desirability bias may lead respondents to over-report adherence to a behaviour they believe they are expected to practise [31–33]. A study in Ghana’s Northern and Upper West regions found that only 17% of individuals with ITN access used a net at any time during the night [4]. Killian et al. [34] found an 11% gap between reported use and access in northern Nigeria; in the south the gap was 36%. Other studies have documented events such as funerals and weddings during which participants consider ITN use impractical or socially unacceptable [5].

The data presented here suggest another possible limitation: complex net use patterns difficult to capture adequately with population-based surveys. Repeated entries to and exits from a net throughout the night may increase exposure to infective bites for those who have exited while allowing mosquitoes to enter and infect those inside. Repeated entries and exits may also increase the likelihood of an occupant leaving the net unsecured, particularly if he or she is a child, is drowsy after waking up in the middle of the night, or is exiting or re-entering in the dark. Late first entry relative to vector biting patterns, sleeping outside a net for part of the night, or sleeping with some part of the body outside, would also limit the net’s effectiveness. A survey that asks respondents whether they slept under a particular net the previous night would likely miss these limitations: in all cases, the respondent could truthfully answer “yes”, and in all cases that answer would provide an incomplete picture of protection.

The positive association between number of people or age groups sharing a net and number of entries and exits is important because in many malaria-endemic countries multiple people share a sleeping space [35]. This includes adults sleeping with children or grandchildren and multiple children sleeping together. This may mean added risk for pregnant women and children under five especially, since individuals in these two groups rarely sleep alone and often sleep with two or more additional people [36, 37].

### Study limitations

Several limitations make it difficult to quantify the potential impact on malaria risk of the ITN use patterns described here. Since the primary purpose of this study was to understand net use in general rather than track entry and exit in particular, some observation data were incomplete or ambiguous about the precise time at which a given entry or exit occurred. This limits the study’s ability to provide more complete measures of time spent outside the net. Some observers tracked net entries and exits more assiduously than others, so some such events likely remained unrecorded. Further, observers had difficulty monitoring more than four sleeping spaces simultaneously and thus likely missed some entries and exits in large households. Overall, data reported here probably underestimate entries and exits.

The same applies to participants securing net borders. Observers were instructed to record this once, when people first entered their nets, but could not always see whether net occupants re-secured net borders upon exiting and later re-entering. Since the investigators did not anticipate multiple entries and exits prior to the study, observers were not instructed to watch for re-securing of nets. Thus, while the data are insufficiently precise to estimate what percentage of the time nets were secured, it seems reasonable to hypothesize that they were often not secured after late night exit or (re)entry.

The risk of the human ITN use patterns described here would depend in part on entomological inoculation rate (EIR) among other vector characteristics [38]. With low EIR, impact could be negligible; with high EIR, it could be significant. Local anopheline feeding behaviour would also have an impact. The *Anopheles darlingi* biting period is unusually long, but higher transmission intensity elsewhere in the world might compensate for the shorter anopheline biting peaks found there [29].

## Conclusion

Despite the limitations, the data on human ITN use patterns presented here are sufficiently suggestive to warrant further exploration. Finer techniques for assessing net use seem warranted, particularly in areas of residual transmission, where even universal ITN distribution campaigns have not produced the expected reductions in malaria transmission or parasitaemia. One option would be to combine night-time direct observation as described here with entomological collections. However, night-time observations require many person hours, are costly and represent a significant burden for both observers and observed household members. An alternative might be a sensor capable of measuring net entries and exits without an observer’s presence. In a recent paper, Krezanoski and colleagues report developing such a sensor and finding in a small pilot study that Ugandan mothers of children under five were willing to test it [39]. If proven effective and socially acceptable, technology of this sort could make quantifying net entry and exit much more precise and economical. Meanwhile, the findings presented here suggest that caution is warranted when interpreting the significance of answers to the question, “Did you sleep under a mosquito net last night?”

## Abbreviations

EIR: entomological inoculation rate
ITN: insecticide-treated bed net
MIS: malaria indicator surveys
SD: standard deviation
USAID: United States Agency for International Development
WHO: World Health Organization
